# Flavonoid Profile of Saskatoon Berries (*Amelanchier alnifolia* Nutt.) and Their Health Promoting Effects

**DOI:** 10.3390/molecules181012571

**Published:** 2013-10-11

**Authors:** Tunde Juríková, Stefan Balla, Jiri Sochor, Miroslav Pohanka, Jiri Mlcek, Mojmir Baron

**Affiliations:** 1 Institute for Education of Pedagogics, Faculty of Central European Studies, Constantine the Philosopher University in Nitra, Drazovska 4, SK-949 74 Nitra, Slovak Republic; E-Mail: sballa@ukf.sk; 2 Department of Viticulture and Enology, Faculty of Horticulturae, Mendel University in Brno, Valtická 337, CZ-691 44 Lednice, Czech Republic; E-Mails: sochor.jirik@seznam.cz (J.S.); MojmirBaron@seznam.cz (M.P.); 3 Faculty of Military Health Sciences, University of Defence, Trebesska 1575, CZ-500 01 Hradec Kralove, Czech Republic; E-Mails: miroslav.pohanka@gmail.com; 4 Karel English College in Brno, Sujanovo namesti 356/1, 60200 Brno, Czech Republic; 5 Department of Food Analysis and Chemistry, Faculty of Technology, Tomas Bata University in Zlin, Namesti T. G. Masaryka 275, CZ-762 72 Zlin, Czech Republic; E-Mails: mlcek@ft.utb.cz

**Keywords:** *Amelanchier alnifolia*, flavonoids, anthocyanins, proanthocyanidins, flavonols, antioxidant activity, biological activity

## Abstract

Flavonoids are a significant group of secondary metabolites in plants. Many of these compounds are potent antioxidants, being an important part in food products derived from the plants. The current status of research on flavonoid compounds in the fruit of Saskatoon berries (*Amelanchier alnifolia* Nutt.) and their health promoting effects, including recommended utilization, are reviewed. The major classes of flavonoids in the fruit are flavonols (quercetin and rutin), flavanes (proanthocyanidin compounds ranging from dimers through to heptamers and even higher polymers) and finally anthocyanins. The flavonoids represented the group of polyphenols that mostly contributed to the antioxidant activity of Saskatoon berries. High content of the flavoinoids antioxidants in the fruit is responsible for the observed anti-inflammatory, antidiadiabetic and chemo-protective effects.

## 1. Introduction

*Amelanchier alnifolia* Nutt. (Saskatoon berry, Juneberry, or serviceberry, Western Serviceberry, Alder-Leaf Shadbush, Dwarf Shadbush, Western Juneberry) is a deciduous shrub that is a native species of the Northern prairies and plains of North America [1]. The fruit is reported as a berry, but it is actually a pome fruit belonging to in the pome fruit family (*Rosacea*) [2]. The “berries” are sweet and edible, but sometimes quite seedy [3]. Saskatoon fruits have excellent flavour attributes when consumed as fresh fruit or processed foods and have the potential to be an economically important fruit crop (similar to blueberries) in Canada. The economic role is emphasized by a fact that saskatoons can be produced in commercially acceptable amounts in Western Canada [1]. Currently, saskatoon fruits are consumed in North America as a fresh fruit or in processed food products such as pies, jams, and jellies. The most common cultivars used by growers include Honeywood, Martin, Northline, Pembina, Smoky and Thiessen [4].

In the last two decades, the Saskatoon berry has been cultivated in many parts of the world [5] for its suitability for various food products and due to its high content of nutrients and polyphenols [6]. Saskatoon berries have an appearance and taste similar to blueberries, but they have a thicker skin and juice. Fresh fruit contains relatively large amounts of potassium, iron and magnesium [7]. Saskatoons appear to be an excellent source of manganese, magnesium, iron, calcium, potassium, copper and carotene. Besides the metal micronutrients, berries of *A. alnifolia* contain phenolic acids including 3-feruloylquinic, chlorogenic, and 5-feruloylquinic acids [8,9]. Saskatoon berries are especially rich in anthocyanins and flavonoid compounds, including rutin, hyperoside, avicularin and quercetin [4,5,7].

Recent research indicates that Saskatoon berries have higher levels of antioxidants compared to the more common berries such as wild blueberries, strawberries and raspberries [10]. In their review, Ozga *et al.* [11] summarized research on nutrient components in saskatoon fruits. The objective of their study was focused on anthocyanins as potential nutraceuticals. On the other hand, there is no review on flavonoid content of *A. alnifolia* in the current literature. Up to now *A. alnifolia* has been used as an ornamental plant species in Slovak and Czech Republic. Recently, the cultivation of *Amelanchier* combining decorative quality and high biological value of fruit has been gaining in popularity. For the reason, we decided to review the recent research on this neglected species in the territory of Slovakia and Czech Republic.

## 2. Flavonoids of Saskatoon Berries

Currently, increasing attention is being paid by consumers to the lesser known fruits which have unusual flavours and qualities. Many of these fruits are rich in antioxidants, especially anthocyanins and proanthocyanidins [12,13]. The demand for saskatoon fruit is increasing in both domestic and international markets because of their excellent flavour and reported health benefits attributed to the flavonoid content [14,15]. In an example*,* phenolic acids, anthocyanins and a series of proanthocyanidin polymers had been revealed in *A. alnifolia* extracts [6]. The authors studied polyphenolic profile of saskatoon berries from four Finnish cultivars. HPLC-analysis revealed over 30 individual phytochemicals in a saskatoon plant. The main berry components were cyanidin-based anthocyanins (63% of the total phenols), quercetin-derived flavonol glycosides, and hydroxycinnamic acids, especially chlorogenic acid [6]. The polyphenolic composition varied among plant parts and the concentrations of compounds varied among cultivars. The most significant cultivar differences were detected in the accumulation of polyphenols in berries.

**Figure 1:**
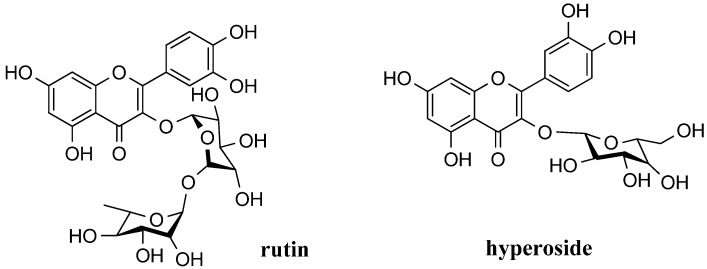
Structures of chosen flavonoid compounds presented in “Smoky” saskatoon fruit.

The major phenolic components in Saskatoon berries are flavonoids [12], including proanthocyanin (PA) [16,17] and anthocyanins [18]. Flavonoid compounds from the fruit include also rutin, hyperoside, avicularin, and quercetin. The total content of the anthocyanin-, flavonol-, and hydroxycinnamate-type phenolics detected in mature “Smoky” saskatoon fruit was 140, 25, and 96 mg/100 g fresh weight, respectively [10]. Structures of the aforementioned compouds are depicted in Figure 1.

ROP *et al.* [19] measured the total flavonoid content in nine cultivars bred in the Czech Republic and Canada. The highest content of flavonoids was examined in the “Smoky” (550.5 ± 20.5 mg of rutin/kg FW), “Thiessen” (531.2 ± 17.7 mg/kg), and above all “Tisnovsky” (562.8 ± 20.0 mg/kg) cultivars. Mazza [20] noticed a similar total flavonoid content value of 530 mg/kg FM in the “Smoky” cultivar. According to results of Juríková *et al.* [21], *Amelanchier* “Tišňovský” and “Smoky”, together with *Morus nigra* “Jugoslavska”, accumulated the highest levels of examined polyphenolic compounds and flavonoids.

On the other hand, most flavonoid identification and quantification studies are limited to mature saskatoon fruit [22,23], with minimal attention paid to developing fruit. Generally, fruit ripening in saskatoon fruit has been categorized into nine maturity stages [24,25]. Fruit maturity stages 5 to 9 are characterized by increasing anthocyanin content.

## 3. Anthocyanins

**Figure 2:**
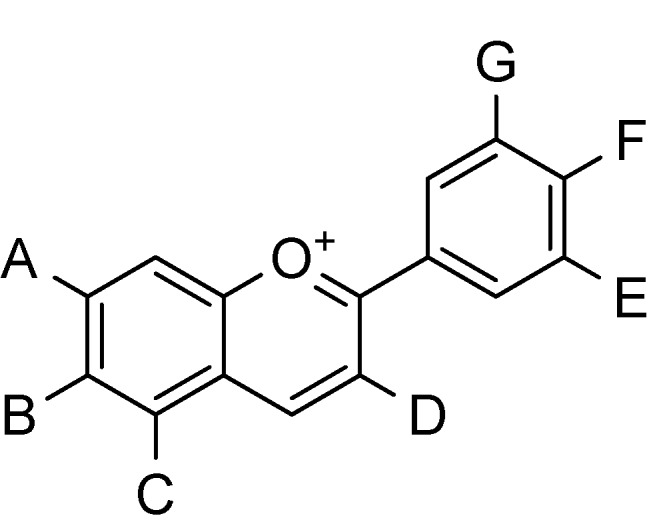
Common structure of anthocyanins. The substituents A–G can be hydrogen, hydroxyl, or methoxyl. Anthocyanins are frequently glycated through hydroxyl substituent D.

Phenolic compounds, particularly the anthocyanins, appear to be the major functional components of blueberries and saskatoon berries. The common structure of anthocyanins is depicted in Figure 2. Values of total anthocyanin content of Saskatoon berries range between 25.1 and 179 mg/100 g of berries fresh weight (FW) [26,27]. The anthocyanidin content reaches a mean value of 111.1 ± 16.8 mg/100 g FW [14]. The total anthocyanin content in four Finnish cultivars varied between 258.7 and 517.9 mg/100 fresh weight [14,22]. The total anthocyanin content (in mg/100 g FW) in saskatoon fruit is comparable to that reported for other small fruits such as blackberries (*Rubus spp*.; 83–326), blueberries (25–495) and bilberries (*Vaccinium myrtillus* L.; 300–320) [28]. On the other hand, the studies of Zatylny *et al.* [4] proved that the anthocyanin content in 16 cultivars of saskatoons was not to as high as in blueberries.

Anthocyanins are more likely to occur in glycoside form [9]. Compared with other fruits at maturity such as blueberry and grape (*Vitis vinifera* L.), which can contain over 20 anthocyanins [28], saskatoons have a relatively simple anthocyanin complement [11].

**Figure 3:**
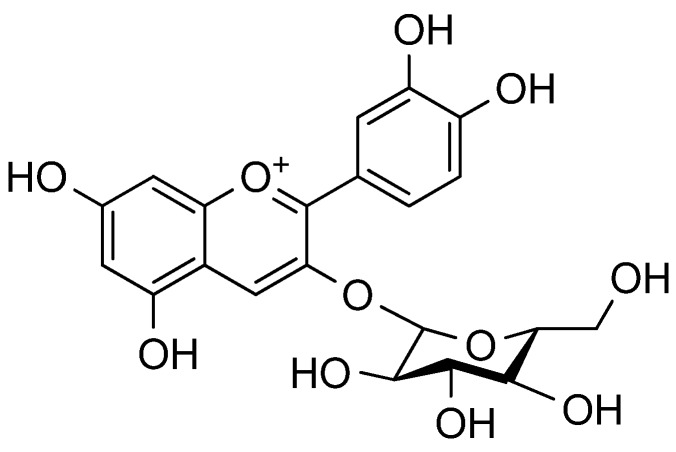
Structure of cyanidin-3-glucoside.

Previous studies on the anthocyanin and flavonol profiles of mature saskatoon fruit have found cyanidin to be a major anthocyanin aglycone. Galactose, glucose, arabinose and xylose are the major sugar units attached at the C3 position of cyanidin in an *O*-glycosylated form [17]. There are at least four anthocyanins in ripe saskatoon berries, of which cyanidin 3-galactoside and 3-glucoside account for about 61% and 21% of the total anthocyanins, respectively [26]. The content of cyanidin-3-galactoside was 115 mg/100 g and that of cyanidin-3-glucoside (Figure 3) 54 mg/100 g [14].

**Figure 4:**
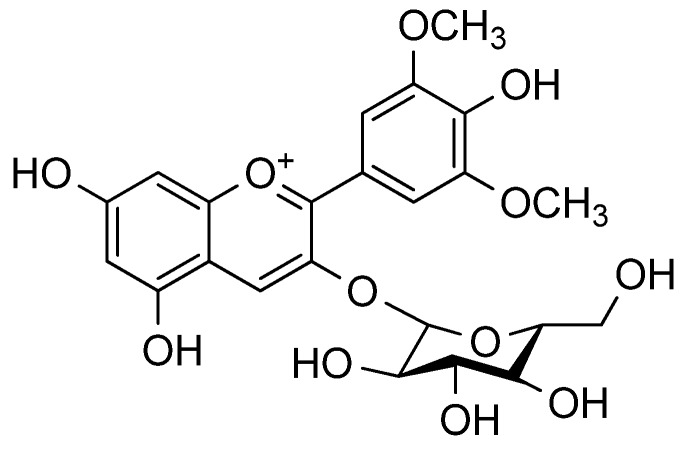
Structure of malvidin-3-glucoside or oenin in some sources.

Hosseinhian *et al.* [29] reported that the Saskatoon berry and wild blueberry in Canada also contained higher amounts of delphinidin 3-glucoside, malvidin-3-glucoside (Figure 4), and malvidin-3-galactoside. Mazza [18] also identified the presence of cyanidin 3-xyloside in the plants. ESI—mass spectral analysis provided the first documentation of petunidin 3-glucoside/galactoside, cyanidin 3,5-diglucoside in Saskatoon berries [30].

Ozga *et al.* [12] characterized the anthocyanins profile of Saskatoon berries using high-performance liquid chromatography, gas chromatography, and liquid chromatography-mass spectrometry. Cyanidin 3-*O*-galactoside, cyanidin 3-*O*-glucoside, cyanidin 3-*O*-arabinoside, and cyanidin 3-*O*-xyloside were identified as the four major anthocyanins in the mature fruit.

**Figure 5:**
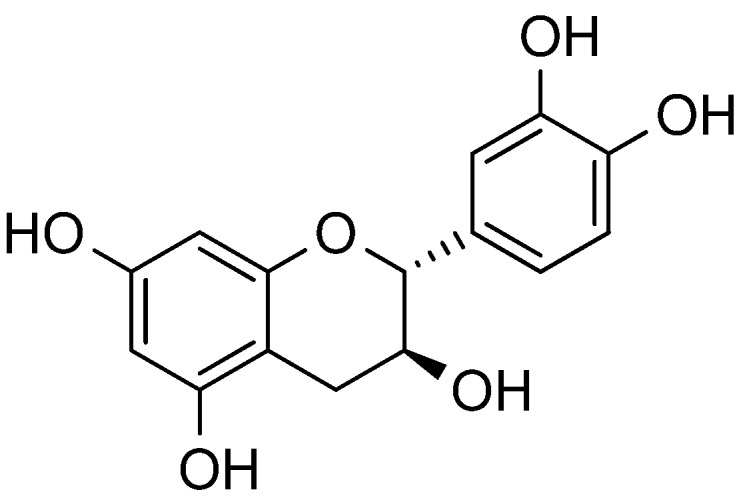
Structure of catechin.

Source, year of production and relative maturity stage of berries at harvest time are important factors influencing total anthocyanin and total phenolic content in the Saskatoon berry fruit [31]. Especially catechin (Figure 5) changes its level as the conditions vary.

Total anthocyanin content was different among cultivars in these studies. Indeed, anthocyanin levels differed in the same cultivars between studies. This might due to differences in growing conditions (including soil type and water availability), year of harvest, and the length of storage period. Very important factor influence the anthocyanins content is the stage of fruit maturity. Indeed, the anthocyanin content of the three major anthocyanins in saskatoon fruit increased twofold from stage 7 to stage 8, and again twofold from stage 8 to stage 9 in cv. “Smoky” [11].

A reduction in anthocyanin content has been observed during cold storage of saskatoon fruit [24,32]. Rogiers *et al.* [32] reported that the greatest reduction in anthocyanin content of saskatoons (cvs. Northline, Smoky, Pembina and Thiessen) occurs within the first 11 days of cold storage at 0.5 °C. Bakowska *et al.* [17] assayed the anthocyanins for 17 different cultivars of Saskatoon berry grown in Canada in fresh fruit and stored by freezing at −20 °C for nine month. Among the assayed cultivars, “Nelson” was the richest in total polyphenols and anthocyanins content (801 mg/100 g FW). Cultivar-dependent changes in polyphenol content were observed after freezer storage at −20 °C for nine month, in the “Lee” cultivar, significant increases in anthocyanins and flavonols occurred, while in “Lee 3” and “Martin” cultivars considerable decreases were observed.

Zatylny *et al.* [4], in accord with the previous study, noticed that the fruit of “Nelson”, in spite of having superior physicochemical properties (the highest content of anthocyanins, high contents of total and soluble solids, high acidity, low SS/TA, low pH), did not have the most appealing flavour when eaten fresh. At the same time, this cultivar was the most suitable for storage by freezing.

High levels of anthocyanins in Saskatoon berries have been associated with many factors that correspond to factors known to stabilize anthocyanins in various fruits, these including high total phenolics, total acids and lower pH along with a low sugars to acids ratio [4,18]. Correlations between anthocyanin content and fruit pH represented (*r* = −0.41, *p* < 0.001) and titratable acidity (*r* = 0.53, *p* < 0.001). These results indicate that fruit with high acidity tended to have higher anthocyanin content. On the other hand, no relationships were found between anthocyanin content, fruit weight and total solids [4]. Fukumoto *et al.* [33] on comparison of several berry extracts also found similar relation between anthocyanins and total phenolic compounds in blueberry and Saskatoon berry (“Smoky”) extracts.

## 4. Proanthocyanidins

Proanthocyanidins (condensed tannins), as the second most abundant group of natural phenolics after lignins, are oligomers and polymers of flavan-3-ol units widely present in, in fruits, berries, nuts and seeds [34,35,36]. Proanthocyanidins, for example, are colorless oligomers and polymers of (flavan-3-ol units) that have demonstrated anti-adhesion, anti-cancer, and antioxidant properties [37]. The degree of polymerization of proanthocyanidins has been shown to influence their activity [38].

Generally, the highest contents of proanthocyanidins per fresh weight were determined in chokeberries, rose hips and cocoa prosucts. Berries and fruits were generally the best sources of proanthocyanidins whereas most of the vegetables, roots and cereals lacked them completely. In berries the considerable variation in proanthocyanidins content was observed [23,38]. Among berries substantial amounts of proanthocyanidins are found in blackthorns, chokeberries, saskatoon berries, blueberries, cranberries, and lingonberries [39,40,41,42,43,44].

It can be mentioned that saskatoon berry proanthocyanidins are very similar to apple procyanidins. As one of the main subclasses of flavonoids, PAs (oligomeric and polymeric flavan-3-ols, also known as condensed tannins due to their brown oxidation color) are widely distributed in many types of fruits, where they provide flavor and astringency to the fruit when consumed [45].

Saskatoon cultivars were also rich in proanthocyanidins: 3% of dry berry biomass and 10%–14% of dry biomass of stems and leaves. Cultivars were observed to be similar in their proanthocyanidin contents [6]. Kraft *et al.* [30] the first time noticed the presence of catechin/epicatechin, epicatechin gallate, and the proanthocyanidin series in this species by ESI—mass spectrophotometry analysis.

The major proanthocyanidins (flavanols) present in the whole fruit, juice, and pulp of strawberry, Saskatoon berry, raspberry, wild blueberry, chokecherry, and seabuckthorn were measured by Hosseinan *et al.* [22]. The extraction and purification were facilitated using flash column chromatography, while separation and identification were accomplished by using HPLC and LC-MS techniques. The highest concentration of proanthocyanidin in juice was found in Saskatoon berry (1,363 mg/100 mL) and the lowest value in strawberry (622 mg/100 mL).

Proanthocyanidin oligomers with different degrees of polymerization were isolated from Saskatoon berries (*Amelanchier alnifolia* Nutt.) by means of gel adsorption and normal-phase liquid chromatography. The proanthocyanidins were identified using electrospray ionization mass spectrometry, nuclear magnetic resonance spectroscopy, and thiolytic degradation coupled with reversed-phase liquid chromatography. The results established that Saskatoon berries proanthocyanidins are essentially of procyanidin type, consisting mainly of epicatechin (2*R*,3*R* derivative of catechin depicted in Figure 5) units linked by B-type bonds [16]. This is in accord with apple and ligonberries samples but in contrast with grape. In grape samples, they are mixtures of procyanidins, partly esterified with gallic acid, and prodelphinidins [46].

Hellstrom *et al.* [23] developed a method for the determination of extractable and unextractable proanthocyanidins to the analysis of several fruit and berry samples. Suitability of the method was tested by analyzing of several plant material including Saskatoon berries. Extractable proanthocyanidins were separated according to their degree of polymerization using normal phase HPLC. Unextractable proanthocyanidins were measured after acid-catalyzed depolymerization as flavan-3-ols (terminal units) and benzylthioethers (external units). Electrospray ionization mass spectrometry (ESI-MS) was used for the identification of proanthocyanidins in the samples. Proanthocyanidin content in Saskatoon berry was 276 mg/100 g; over one-third of these compounds were determined to be unextractable. Extractable proanthocyanidins were represented by P1 (21.4 ± 0.4), P2 (20.2 ± 0.7), P3 (21.7 ± 1.3), P4-6 (46.1 ± 3.7), P7-10 (21.0 ± 1.9) ˃P10 (44.6 ± 4.1), total extractable took 175 ± 12 mg/100 g FW ± standard deviation). Unextractable procyanidins created smaller fraction DP 8.2 ± 0.1, in total 101 ± 9 (FW ± standard deviation).

Hosseinian *et al.* [16] measured the content of extractable proanthocyanidins in Manitoba berries, including Saskatoon. The result reported there in (369 mg/100 g) is somewhat higher than that in the present study. Different cultivars and climatic conditions as well as methodological differences may explain the divergence [47]. The mDP of PAs varied (5 to 13) also among the cultivars studied. The highest level of proanthocyanidins was proved in “Nelson” cultivar. In a total, 278 mg/100 g FW of proanthocyanidins was found in the samples [17].

## 5. Flavonols

**Figure 6:**
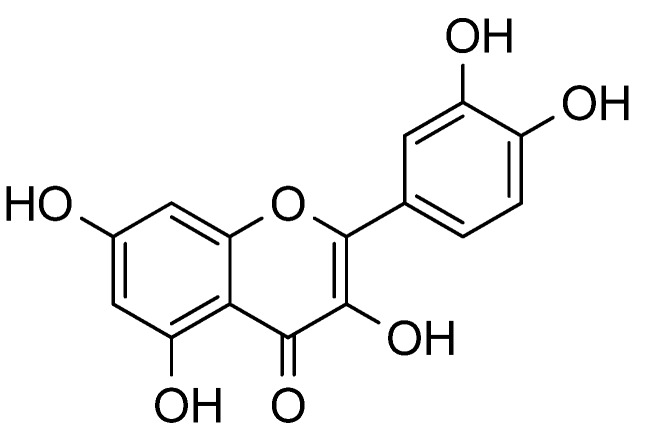
Structure of quercetin.

Quercetin (Figure 6) was found to be the major flavonol aglycone and quercetin 3-*O*-galactoside (Figure 3) was the most abundant flavonol in the fruit of most saskatoon cultivars. Quercetin levels reach up to 16.95 mg/100 g. The major flavonols identified in mature saskatoon fruit include the quercetin-diglycosides (quercetin 3-*O*-rutinoside, quercetin 3-*O*-robinobioside, and quercetin 3-*O*-arabinoglucoside) and the quercetin-monoglycosides (quercetin 3-*O*-galactoside, quercetin 3-*O*-glucoside, quercetin 3-*O*-arabinoside, and quercetin 3-*O*-xyloside [12].

Gazdik *et al.* [48] primarily focused their study on basic electrochemical behavior of rutin and quercitrin in samples of neglected fruit species—Chinese hawthorn, Saskatoon berry and honeyberry. The results displayed higher content of quercitrin in *L. edulis* (220 mg/kg) and *A. alnifolia* (230 mg/kg FW) fruits. Saskatoon berries, showed the highest levels of quercitrin, *(Amelanchier alnifolia* Nutt. Bunge) in cultivars “Martin” 231 ± 1.4 mg/kg (*Amelanchier alnifolia* Nutt. Bunge), “Northline” 233 ± 1.9 mg/kg among studied samples of lesser known fruit species—*Crateagus pinnatifida* Bunge, *Lonicera edulis* “Sinoglaska”. Among another lesser known fruit species quercitrin levels above 100 mg/kg were determined in black mulberry “Jugoslavska” (143 mg/100 g), “Amur” (126 mg/100 g) and “Thiessen” (124 mg/100 g) [21].

**Figure 7:**
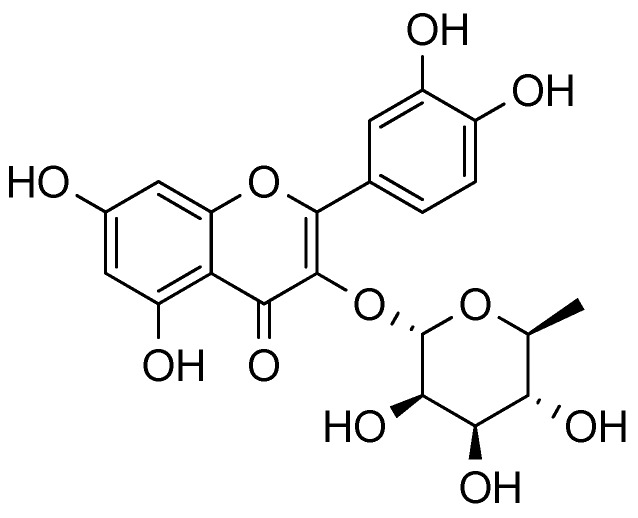
Structure of quercitrin.

Gazdik *et al.* [49] used a HPLC—EDE system for identification and quantitative determination of neuroprotective flavonols—rutin, quercetin, quercitrin (Figure 7) and their glycosides in lesser known fruit species. The results of experiment showed that fruit of *Amelanchier canadensis* had almost four times higher content of neuroprotective flavonols (rutin, quercetin and quercitrin) compared to berries of sea buckthorn and cornelian cherry Results of their experiment showed, that *Amelanchier* berries (*A. canadensis*, *A. alnifolia* “Thiesen” had generally 2–3 times higher content in quercetin glycosides than in berries of sea buckthorn samples (“Buchlovicky”, “Pavlovsky”, “Peterbursky” and “Vitaminova”). Saskatoon berries appears to be similar in rutin content to berries of blue honeysuckle 5–32 mg/100 g FW reported by Jurikova et al [50]. Rich sources of rutin were presented in *A. alnifolia* cultivars “Martin” 34.2 ± 3.7 and “Northline” 20.8 ± 2.1 mg/100 g FW [19].

According to the results from a comparative study of polyphenolic content in lesser known fruit species both analyzed cultivars of Saskatoon berry can be characterized by exceptionally high value of quercetin 23.6 mg/100 g in “Thiessen” respectively 30.7 mg/100 g in “Smoky”. The concentration of rutin in “Thiessen” was two times higher [21] than reported for samples from *Amelanchier* “Martin” [19].

Although the major anthocyanin and flavonol compounds in mature saskatoon fruit have been identified by Ozga *et al.* [12], minimal information exists on the temporal and spatial accumulation patterns of these two classes of flavonoids over fruit development.

Knowledge of the phenolic composition of mature saskatoon fruit, especially anthocyanins, flavonols can help to better understanding of Saskatoon berries antioxidant properties and health promoting activity.

## 6. Biological Activity of Saskatoon Berry Flavonoid

Historically, the native people used Saskatoon berries for many things. A weaker mixture of fruit tonic was also used to ease stomach and liver troubles. Beside problems with the gastrovascular cavity, they treated sore eyes with the berries. The settlers adopted the saskatoon as a medicine as well. Diarrhea was treated with crushed green berries [3]. Due to a high content of antioxidant compounds, the dietary intake of Saskatoon fruit has a positive and profound impact on human health, performance and disease [17].

### 6.1. Antioxidant Activity of Fruit

The antioxidant activity is defined as an ability of the compound (or mixture of compounds) to inhibit oxidative reaction of various biomolecules (e.g., prevent the peroxidation of lipids) [51,52,53,54]. Methods of the antioxidant activity determination are usually based on the direct reaction of the studied molecule with radicals (scavenging) or on the reaction with ransition metals [55,56,57,58]. The current research is very scant in terms of the antioxidant activity of Saskatoon berries. It is important to print out that vitamin C is not present in any appreciative amounts in Saskatoon berries fruits [59]. The aforementioned antioxidant capacity of Saskatoon berries does not come from ascorbic acid, contrary to the findings in other similar soft fruit. For this reason, different biological effects during the course of the berries consumption can be anticipated when compared to consumption of other fruits containing high amounts of ascorbic acid.

The results of Fukumoto *et al.* [33] suggested that fruit extract has both prooxidant and antioxidant activity. In the study by Wang *et al.* [60], it was reported that anthocyanin in form of rich crude extracts of phenolic compounds from several berries (Saskatoon berries, blueberries, blackberries and black currants) is able to impair nitric oxide (NO) production. Ripe berry juice was said to to relieve upset stomach. When boiled, it was used as ear drops against problems due to inflammation [61]. Its potential health benefits are similar to blueberries and cranberries [62].

The current studies have showed that two cultivars of Saskatoon berries, “Thiessen” and “Smoky”, possess a high radical scavenging activity attributed to the presence of anthocyanin in the phenolic fractions and their extracts suppressed peroxy-radical intracellular oxidation. Cultivar “Thiessen” exhibited higher antioxidant activity compared to cultivar “Smoky” due to its higher anthocyanins content. Total anthocyanins content significantly correlated to free radical scavenging activities [15]. According to Bakowska *et al.* [3], cultivar “Nelson” exhibited the highest antioxidant activity measured by DPPH and ABTS radical tests (2.8 and 5.0 mM/100 g FW). During the freezer storage, the antioxidant activity remained unchanged except for the “Smoky” that showed to be the most sensitive cultivar during storage. On the other hand, Nelson and Lee 2 were the most stable cultivars during the storage.

In the lipid peroxidation inhibitory assay, the anthocyanin mixture at 10 ppm presented activity of 72% compared to 89%, 87% and 98% for commercial antioxidants, butylated hydroxyanisole, butylated hydroxyanisole, butylated hydroxytoluene and *tert*-butylhydroxylquinone at 1.67; 2.2; and 1.67 ppm respectively. At 10 ppm, cyaniding-3-galactoside, cyanidin-3-glucoside and cyanidin inhibited lipid peroxidation in a range70% to 78% [14].

Gazdik *et al.* [49] obtained flavonoid-rich samples from some less common fruit species—Blue Honeysuckles (*Lonicera Kamtschatica* and *Lonicera edulis*, Turcz. ex. Freyn), Saskatoon berry (*A. alnifolia* Nutt.) and Chinese Hawthorn (*Crataegus pinnatifida* BUNGE)—grown in the Czech Republic and then characterized them in terms of biological value based on the results from a relative antioxidant capacity assessment. The antioxidant content evaluation was based on the total flavonoid amount, determined by liquid chromatography with electrochemical detection (HPLC-ED). A DPPH^•^ test was applied as a reference. The antioxidant content measured in Chinese Hawthorn fruit extract identified it as a potent source of flavonoid antioxidants, with a content 9-fold higher than that seen in *Amelanchier* fruit. Furthermore, the high correlation coefficient between total flavonoid content and antioxidant capacity (*R*_2_ = 0.8870; *y* = 156.52*x* − 228.53) was observed [19].

### 6.2. Anti-Inflammatory Activities

More recently, Wang *et al.* [19] found that a concentrated crude extract of *A.alnifolia* berries inhibited nitric oxide production in activated macrophages, indicating a potential protective role against chronic inflammation. Generally, all health promoting activities including anti-inflammatory activity of berries are related to high anthocyanins and proanthocyanidins content [19]. Moreover, Kay *et al.* [63] have confirmed that humans have the high capacity to metabolize cyanidin 3-glycosides, present in fruit including saskatoon berries and blueberries. Concentrations of anthocyanins and anthocyanin metabolites in the urine reached levels of 8.1 µg/mL within 5 h post-consumption and persisted for 24 h in urine samples at levels of 5–6 ng/mL. In addition, total levels of anthocyanins and anthocyanin metabolites detected in the serum were observed at a concentration of 312.4 ng/mL within 2 h post consumption. Cyanidin 3-galactoside accounted for 55.8% (4.46 µg) and 64.7% (202.24 ng) of the detected anthocyanins, in the urine and serum samples respectively. The metabolites consisted of glucuronide conjugates as well as methylated and oxidized derivatives of cyanidin 3-galactoside and cyanidin 3-glucuronide. Therefore, the complement of anthocyanins in saskatoon fruit appears well suited to contribute health benefits [11].

At 100 ppm, the anthocyanin mixture inhibited cyclooxygenase 1 (COX-1) and COX-2 enzymes at 66 and 67%, respectively. Anthocyanins inhibited COX-1 enzyme 50.5, 45.6 and 96.4% respectively at 100 ppm, whereas COX-2 inhibition was the highest for cyanidin at 75% [14]. From the aforementioned, significant impact on inflammatory processes due to COX-2 and blood clotting due to COX-1 can be anticipated in people with increased alimentation by the berries. Unfortunately, exact results from a clinical investigation are still missing.

Bovin *et al.* [64] investigated the potential anti-inflammatory effects of Saskatoon berry juices in relation to other berry crops. They tested ability of juices to inhibit TNF-induced expression of COX-2 in PC-3 cells. Results of experiment showed that several berry juices inhibited the expression of COX-2 and can be ranked according to their inhibitory potency as: gooseberry (83%) > blackberry (73%) = sea buckthorn (73%) = cranberry (73%) > raspberry (43%) = black currant (43%) > white currant (49%) > serviceberry (38%) > high-bush blueberry (21%) > velvet-leaf blueberry (14%).

### 6.3. Antitumor Activity

Wang *et al.* [60] demonstrated that phenolic compounds, especially flavonoids found in blueberries and saskatoon berries, inhibited nitric oxide (NO) production in bacterial lipopolysaccharide/ interferon-γ activated RAW 264.7 macrophages. Beside this, blueberry and saskatoon berry anthocyanin-rich extracts both induced tumour necrosis factor α (TNF-α) production and acted as modulators of the immune response in activated macrophages [62]. This research provides evidence that phenolic compounds in saskatoon berries and blueberries are able to reduce the oxidative stress mediated by NO. Suitability of the compounds for protection against cardiovascular and chronic inflammatory diseases is discussed.

Boivin *et al.* [64] studied potential chemopreventive activities of edible berries. Strawberry, raspberry, black currant, red currant, white currant, gooseberry, high-bush blueberry, low-bush blueberry, velvet leaf blueberry, serviceberry, blackberry, black chokeberry, sea buckthorn and cranberry were evaluated for antioxidant capacity, anti-proliferative activity, anti-inflammatory activity, induction of apoptosis and cell cycle arrest.

The growth of various cancer cell lines, including those of stomach, prostate, intestine and breast, was strongly inhibited by raspberry, black currant, white currant, gooseberry, velvet leaf blueberry, low-bush blueberry, sea buckthorn and cranberry juice. Serviceberry juice was also a weak inhibitor of cancer cell proliferation (0%–30% inhibition at 50 µL/mL). On the other, the tested juice was very rich for antioxidant capacity (14.6 µmol TE/mL), extrapolation of the results to other flavonoid containing food and beverages can disputable for the reason. Beside this, no correlation was found between the anti-proliferative activity of berry juices and their antioxidant capacity (*p* > 0.05).

### 6.4. Hypoglycemic and Antidiabetic Effect

Anthocyanins and proanthocyanidins fraction of fruit act as hypoglycemic agents and strong inhibitors of IL-1β and COX-2 gene expression. Berries also exhibited the activity to regulate lipid metabolism (LIM, 2012). Fruit from the related species, *A. canadensis* and *A. arborea*, also inhibited cyclooxygenase-1 and -2 *in vitro*, indicating a role in moderating inflammation [14].

Kraft *et al.* [30] examined the antidiabetic activity of Saskatoon berries. The non-polar fraction of *A. alnifolia* strongly inhibited an enzyme involved in the etiology of diabetic microvascular complications, aldose reductase, with a proved inhibition of 82%. Polar berry fraction reduces expression of IL-1β (one of the anti-inflammatory markers) and also improves glucose uptake via an insulin-like effect. The crude extract and polar fraction, on the other hand, exhibited significant capacity to improve glycogen accumulation at a basal state. Screening through a series of lipid metabolism biomarkers (fatty acid oxidation and energy expenditure) further showed that these berries had the ability to modulate lipid metabolism and energy expenditure consistent with amelioration of metabolic syndrome. Saskatoon berry with a series of proanthocyanidin polymers, suggesting that higher molecular weight proanthocyanidins may be integral to the improved glycogen accumulation [65]. Zhang *et al.* [66] identified the potential antidiabetic mechanism of serviceberry. Serviceberry plant samples consisting of leaves, twigs, and leaves with berries were extracted and fractionated. Ethyl acetate and water fractions were tested for inhibition of α-glucosidase activity *in vitro*. Diet-induced obese, hyperglycemic C57Bl6 mice were administered serviceberry leaf extract prior to sucrose-, starch-, or glucose-loading to test for α-glucosidase inhibition and decreased post-prandial glycemic response. In the course of screening for potential antidiabetic mechanisms, serviceberry leaf extracts and subfractions demonstrated potent inhibitory activity against mammalian intestinal α-glucosidase activity (EC 3.2.1.20).

## 7. Conclusions

Understanding the polyphenolic profile of lesser-known fruit species can stimulate an interest in maximizing their nutritional effects in human diet. The main fraction of flavonoids responsible for their health promoting activities are anthocyanins and proanthocyanidins. Proanthocyanids represent compounds influence lipid metabolism and regulate level of glucose and glycogen accumulation. Saskatoon berry anthocyanin-rich extracts both induced TNF-α and have anti-inflammatory effects. We can conclude that Saskatoon berry has perspective potential in anticancer therapy and as an important antidiabetic agent.
